# Clinical burden of community-associated infections caused by multidrug-resistant Pseudomonas aeruginosa: a propensity-matched longitudinal cohort study in Southern China

**DOI:** 10.3205/dgkh000506

**Published:** 2024-10-23

**Authors:** Mouqing Zhou, Baohua Xu, Zhusheng Guo, Yongfeng Zeng, Jiayao Lei, Evangelos I. Kritsotakis, Jiancong Wang

**Affiliations:** 1 Department of Infection Control, DongGuan SongShan Lake Tungwah Hospital, DongGuan, Guangdong Province, China; 2 Department of Science Research, and Education, DongGuan Tungwah Hospital, DongGuan, Guangdong Province, China; 3 Department of Microbiology, DongGuan Tungwah Hospital, DongGuan, Guangdong Province, China; 4 Department of Infection Control, DongGuan Tungwah Hospital, DongGuan, Guangdong Province, China; 5 Department of Medical Epidemiology and Biostatistics, Karolinska Institutet, Stockholm, Sweden; 6 Laboratory of Biostatistics, Division of Social Medicine, School of Medicine, University of Crete, Heraklion, Greece; 7 Institute of Biometry and Epidemiology, German Diabetes Center, Heinrich Heine University Düsseldorf, Düsseldorf, Germany

**Keywords:** community-associated infections, Pseudomonas aeruginosa, multidrug-resistant pathogens, incidence density, age- and sex-specific, China, Dongguan

## Abstract

**Background:**

Limited research has been conducted on the burden of community-associated infections caused by multidrug-resistant *Pseudomonas aeruginosa* (CA-MDRPa). We quantitatively modeled the incidence rate and clinical factors associated with CA-MDRPa among hospitalized patients in Southern China.

**Methods:**

Data were obtained from the local nosocomial surveillance system. Poisson regression was applied to estimate annual incidence rate ratios (IRRs) from 2018 to 2021. After propensity-score 1:2 matching, multivariable conditional logistic regression was used to identify factors for CA-MDRPa upon admission and adverse clinical outcomes during hospitalization.

**Results:**

278 patients were clinically and microbiologically diagnosed with CA-MDRPa and 647 with CA-non-MDRPa. CA-MDRPa rate exhibited a slight, non-significant, increase during the research period (IRR=1.03; 95% confidence interval [CI], 0.93–1.15). Neurological conditions, cardiovascular diseases, respiratory disorders, urinary tract infections, and use of cefoperazone/sulbactam prior to admission were identified as risk factors for CA-MDRPa upon admission. CA-MDRPa upon admission was associated with ESBL-producing *P. aeruginosa* acquisition during hospitalization (odds ratio [OR], 2.70; 95% CI, 1.53–4.77) and increased in-hospital mortality (OR, 2.24; 95% CI, 1.17–4.28).

**Conclusions:**

The findings emphasize the importance of regular targeted screening for CA-MDRPa upon hospital admission and offer valuable insights for strengthening infection control and antimicrobial stewardship programs.

## Zusammenfassung

**Hintergrund:**

Zur Belastung durch Community-assoziierte Infektionen, die durch multiresistente *Pseudomonas aeruginosa* (CA-MDRPa) verursacht werden, ist die Datenlage limitiert. Daher wurden die Inzidenzrate und klinische Faktoren im Zusammenhang mit CA-MDRPa bei hospitalisierten Patienten in Südchina quantitativ modelliert.

**Methode:**

Die Daten stammen aus dem lokalen nosokomialen Surveillancesystem. Zur Schätzung der jährlichen Inzidenzratenverhältnisse (IRRs) von 2018 bis 2021 wurde eine Poisson-Regression angewandt. Nach einem Propensity-Score-Matching im Verhältnis 1:2 wurde eine multivariable logistische Regression verwendet, um Faktoren für CA-MDRPa bei der Aufnahme und nachteilige klinische Ergebnisse während des Krankenhausaufenthalts zu ermitteln.

**Ergebnisse:**

Bei 278 Patienten wurde CA-MDRPa und bei 647 mit CA-non-MDRPa klinisch und mikrobiologisch diagnostiziert. Die CA-MDRPa-Rate wies im Untersuchungszeitraum einen leichten, nicht signifikanten Anstieg aufs1,03; 95% Konfidenzintervall 0,93–1,15). Neurologische Erkrankungen, kardiovaskuläre Erkrankungen, Atemwegerkrankungen, Harnweginfektionen und die Verwendung von Cefoperazon/Sulbactam vor der Aufnahme wurden als Risikofaktoren für CA-MDRPa bei der Aufnahme identifiziert. Das Vorhandensein von CA-MDRPa bei der Aufnahme war mit dem Erwerb von ESBL-produzierenden *P. aeruginosa* während des Krankenhausaufenthalts (Odds Ratio [OR], 2,70; 95% Konfidenzintervall 1,53–4,77) und erhöhter Sterblichkeit im Krankenhaus (OR, 2,24; 95% CI, 1,17–4,28) assoziiert.

**Schlussfolgerungen:**

Die Ergebnisse unterstreichen die Bedeutung eines regelmäßigen, gezielten Screenings auf CA-MDRPa bei der Krankenhausaufnahme und eröffnen wichtige Erkenntnisse zur Infektionsprävention und zum Antibiotika Stewardship.

## Introduction

Multidrug-resistant *Pseudomonas aeruginosa* (MDRPa) is a deadly and difficult-to-treat pathogen that significantly impacts patient care globally [1], [2]. The World Health Organization (WHO) has labeled carbapenem-resistant *P. aeruginosa* as a critical priority pathogen, urging the research and development of new antibiotics [3]. Besides drugs, the WHO also recommends that countries implement national action plans to contain antimicrobial resistance in order to prevent the spread of MDRPa and reduce the incidence of the disease not only in hospitals but also within the community [4]. Prospective epidemiological surveillance – a vital national-level component of a national action plan for infection prevention and control [4], [5] – can provide real-time understanding of the epidemiological dynamics of MDRPa development [6]. The European Antimicrobial Resistance Surveillance Network (EARS-Net), for example, has a long history of collecting and reporting healthcare-associated infections caused by MDRPa, using invasive clinical culture samples from participating hospitals across Europe [7]. Healthcare stakeholders and infection-control specialists at the hospital level in Europe are thus informed of the pathogen’s resistance dynamics by comparing annual in-hospital incidence data [7].

However, based on Medline (via PubMed) searches, there appears to be limited research on the burden of community-associated infection caused by MDRPa (CA-MDRPa) – an investigatory task that is particularly challenging [8]. Incidence surveillance and monitoring are quite resource-demanding, which increases the complexity of both the surveillance of outpatient clinical prescriptions of antibiotics in the community and the monitoring of the accessibility of antibiotics in pharmacies [9]. The Chinese Ministry of Health has implemented policies and surveillance systems to promote the rational use of antibiotics and to monitor resistance patterns [10]. However, despite these efforts, combining data from outpatient prescriptions and pharmacies to reflect actual antibiotic consumption in the community is challenging without an advanced digital surveillance system that connects outpatient clinics and pharmacies [11]. Furthermore, laboratory isolation of MDRPa in outpatient clinics for asymptomatic carriers is not regularly conducted, which is partly due to the limited availability of labor resources and lab technicians [12]. All these issues are compounded by a limited understanding of the epidemiology of MDRPa when acquired in the community in China, particularly in the context of the ongoing discussions on resistance from environmental sources as conceptualized in the One Health framework [13]. 

Given the challenges of community surveillance of MDRPa, hospital-based monitoring of CA-MDRPa is particularly important. Hospital infection-control specialists have raised substantial concerns about suspected CA-MDRPa carriers, warning that if they are not identified and screened upon admission then they are more likely to contaminate hospital environments through daily contact with patients and doctors [14], [15], thus increasing the chance of patient-to-patient transmission, especially without proper isolation of high-risk patients and adequate hospital disinfection practices [16]. Such carriers are a potential source of preventable hospital-related MDRPa outbreaks, which can ultimately lead to increased medical expenditures and in-hospital mortality [15]. Therefore, to prepare epidemiological reference data to support the development of in-house admission screening protocols for *P. aeruginosa* at our institution [17], we analyzed hospital-wide registry data that encompassed patients diagnosed with either CA-MDRPa or community-associated infection caused by non-MDRPa (CA-non-MDRPa) from 2018 to 2021 at a university-affiliated hospital in Southern China.

The specific aims of the current study were

1. to perform quantitative analyses and modeling of the incidence density of CA-MDRPa and CA-non-MDRPa;
2. to delineate the epidemiological patterns of age- and sex-specific acquisition of these infections by pooling all analyzed data;
3. to examine the potential factors influencing CA-MDRPa among hospitalized patients upon admission in comparison with CA-non-MDRPa, using propensity scores to balance the two groups for multivariable analysis; and
4. to assess the association between CA-MDRPa upon admission (as an independent covariate) and the risk of developing ESBL-producing *P. aeruginosa* during hospitalization and in-hospital mortality.

## Material and methods

### Study design

The study had a hospital-wide longitudinal cohort design and was based on prospectively collected registry data from the Dongguan Nosocomial Infection Surveillance System [6]. Access to the dataset for Dongguan Songshan Lake Tungwah Hospital was granted based on the stated research purpose, and the project received full support from both the hospital’s leadership and the infection-control department.

### Study population 

The study population comprised all hospitalized patients admitted to the hospital, across all ages and departments, whose admission date was on or after January 1, 2018, and whose discharge date was on or before December 31, 2021. Clinical culture isolations (such as from sputum, urine, or blood) from the patients were microbiologically analyzed, and those diagnosed with *P. aeruginosa* infections by physicians were included in the research dataset.

### Data collection 

Demographic information, infection and clinical data were collected, which included age, sex, admission/discharge date, admitting departments, admission diagnoses, infection sites, length of stays, comorbidities related to diabetes or immunocompromised conditions, use of cefoperazone/sulbactam or piperacillin/tazobactam prior to admission, development of extended-spectrum beta-lactamase (ESBL)-producing *P. aeruginosa* during hospitalization, and in-hospital mortality. All these data were retrieved from the Dongguan Nosocomial Infection Surveillance System, which directly links to the patients’ medical records. Infection-control specialists checked and verified the accuracy of the data. 

### Definitions

Community-associated infections were defined as those diagnosed within 48 hours of admission in patients who had not recently interacted with healthcare services at other hospitals or clinics – for example, those who had not received hemodialysis or other medical services and who were not transferred from another hospital in the previous 30 days [18], [19]. Applying the criteria of the EARS-Net surveillance protocol, we removed duplicate data by isolating *P. aeruginosa* from the first clinical sample – specifically, the first infection diagnosed [7]. Such infections were considered initial *P. aeruginosa* infections regardless of subsequent culture isolations within 48 hours of admission [8], [19].

All the microbiological tests were performed according to the US National Clinical and Laboratory Standards Institute guidelines [6]. MDRPa was defined as a *P. aeruginosa* strain showing non-susceptibility to at least one antimicrobial agent in three or more antimicrobial groups [20]. Conversely, non-MDRPa was defined as a strain that did not meet this criterion, i.e., showing non-susceptibility in no more than two antimicrobial categories [19]. The development of ESBL-producing *P. aeruginosa* during hospitalization was defined as the acquisition or expression of ESBL enzymes by *P. aeruginosa* strains isolated from a patient while admitted to a healthcare facility [21].

### Health outcome 

The primary study outcome was occurrence of CA-MDRPa and CA-non-MDRPa upon hospital admission. Secondary outcomes included acquisition of ESBL-producing *P. aeruginosa* during hospitalization and in-hospital mortality.

### Statistical analysis 

#### Poisson regression model for the incidence rate ratio 

The absolute numbers of newly diagnosed CA-MDRPa and CA-non-MDRPa cases were aggregated monthly from January 2018 to December 2021. We calculated the incidence densities at monthly intervals by dividing the total numbers of CA-MDRPa and CA-non-MDRPa cases each month by the total number of hospital patient admissions for that month. These rates were then expressed per 100 patient admissions. To model the incidence trends of CA-MDRPa and CA-non-MDRPa, we used a Poisson regression model, which included the natural logarithm of the number of hospitalized patient admissions as an offset and estimated the average annual relative changes in incidence from 2018 to 2021 expressed in terms of incidence rate ratios. The model’s effectiveness was evaluated by examining its summary, including the dispersion parameter and 95% confidence intervals (CIs). These metrics allowed statistical significance and the impact of time on incidence rates to be assessed. We also separately modeled the incidence trends for two major types of community-associated infections – lower respiratory tract infections and urinary tract infections – caused by MDRPa and by non-MDRPa.

#### Propensity-score matching 

To ensure balanced comparisons between the CA-MDRPa and CA-non-MDRPa groups, we employed propensity-score matching using a logistic regression model to estimate propensity scores, treating age, sex, and admitting departments as covariates. We used the *MatchIt* R package for 1:2 matching of the CA-MDRPa and CA-non-MDRPa groups using nearest-neighbor matching with a maximum caliper width of 0.2 times the standardized difference of the logit of the propensity score [22], [23], [24] (Supplementary Material 1 in Attachment 1). Subclass values were created to identify matched pairs or groups from the propensity-score analysis, balancing the distribution of covariates between the CA-MDRPa treatment group and the CA-non-MDRPa control group.

We also generated propensity-score density plots to graphically evaluate the matching quality by comparing the overlaps and similarities in the distributions of the propensity scores between the two groups. Unmatched data were not considered for further analysis. 

#### Variable selection for multivariable analyses

The basis for variable selection was not only subject-matter information, as presented in the literature and systematic reviews, but also the availability of data from the Dongguan Nosocomial Infection Surveillance System [1], [2]. To ensure reliable estimation in the logistic regression analysis, we followed the criteria suggested by Peduzzi et al. [24], [25] who recommended that the number of events per variable be ten or greater to avoid biased regression coefficients in both the positive and negative directions.

#### Multivariable analysis using a conditional logistic regression model

To account for potential confounding covariates and ensure reliable estimation, we first used the *clogit* function from the *Survival* R package to conduct a multivariable analysis on the propensity-score-matched dataset with a conditional logistic regression model (Supplementary Material 2 in Attachment 1) [24]. In the model, CA-MDRPa was treated as the dependent variable, while age, sex, admitting department, admission diagnoses, infection sites, comorbidities, antibiotic use prior to admission, and admission during the COVID pandemic were treated as independent variables. The subclass variable was used as a stratification factor to account for matched pairs or groups. Statistical significance was defined as a two-tailed *p*-value of less than 0.05. Results from the conditional logistic regression model are presented as odds ratios (ORs) with corresponding 95% CIs. Furthermore, a mixed-effects logistic regression model was fitted using the *glmer* function from the *lme4* R package. We finally compared the conditional logistic regression model with the mixed-effects logistic regression model using evaluation metrics such as the Akaike information criterion, Bayesian information criterion, and Log-likelihood value to assess the robustness and fitness of the models [24].

We also used multivariable analysis, again with a conditional logistic regression model, to assess the impact of CA-MDRPa and other clinical factors – treated as independent variables – on the probabilities of two important clinical adverse events: the development of ESBL-producing *P. aeruginosa* during hospitalization and in-hospital mortality, treated as dependent variables.

None of the study variables had missing data, and all the analyses were performed and graphs prepared using the statistical software R, version 4.3.2 (The R Foundation for Statistical Computing Platform). We also reported the age- and sex-specific distributions of absolute cases of CA-MDRPa and CA-non-MDRPa, consistent with the EARS-Net surveillance report [7].

### Ethics 

This study received ethical approval from the Ethics Committee of Dongguan Songshan Lake Tungwah Hospital (Reference number: SDHKY-2024-005-01). Because the data originated from the local nosocomial surveillance system and did not include any individually identifying patient information, written informed consent from patients was not required. This study was reported in accordance with the Strengthening the Reporting of Observational studies in Epidemiology (STROBE) statement [26] (Supplementary material 3 in Attachment 1).

## Results

### Study population 

**Figure 1:**
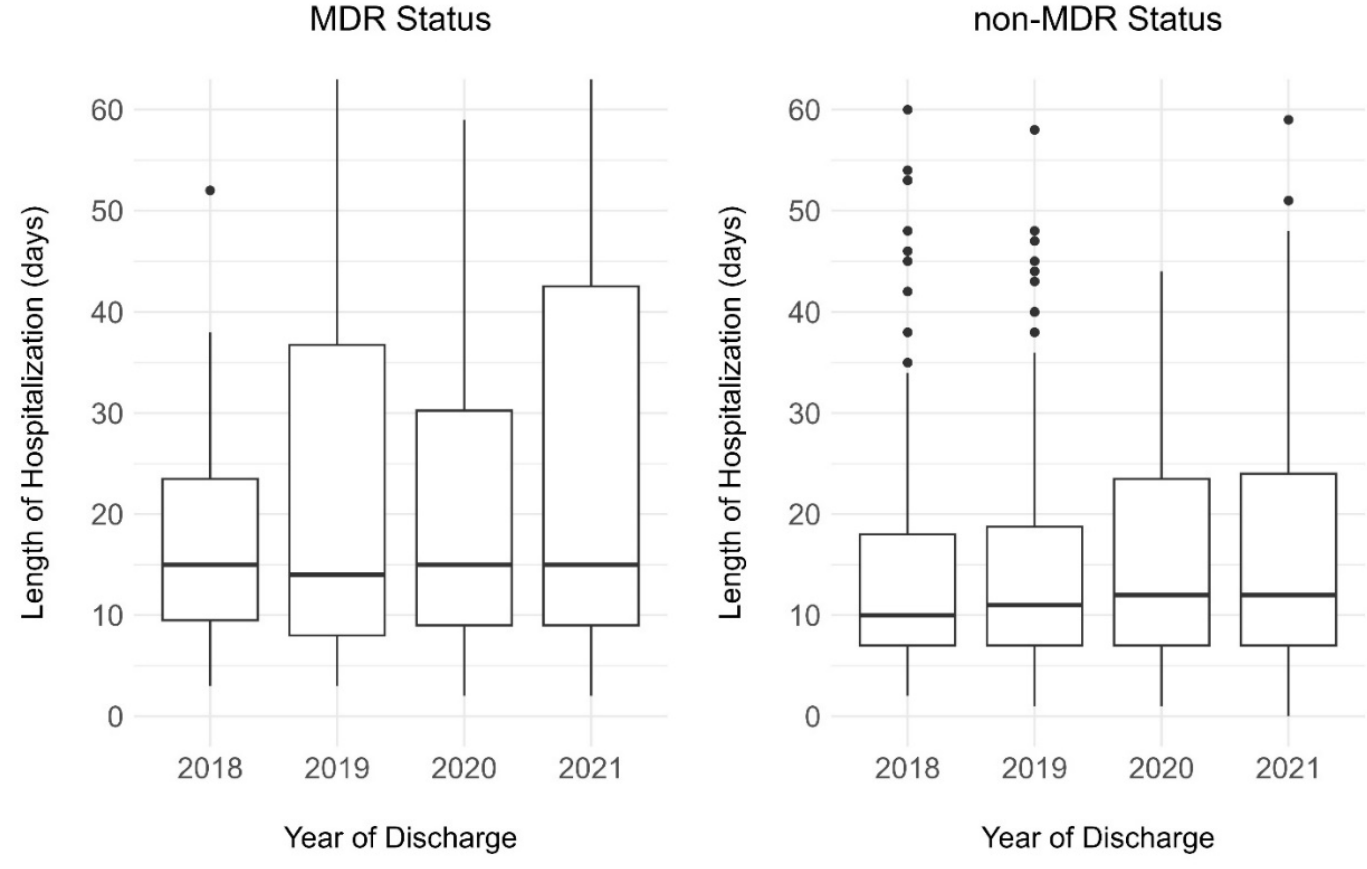
Length of hospitalization by MDR status and year, 2018–2021

Following data deduplication, we found that, between 2018 and 2021, a total of 278 patients were clinically and microbiologically diagnosed with a CA-MDRPa and 647 patients with a CA-non-MDRPa within two days of admission. During the same period, there were 435,713 patient admissions, accumulating a total of 3,200,268 patient days. The median age of patients with CA-MDRPa during this period (62 years, interquartile range [IQR]: 50–71) was higher than the median of patients with CA-non-MDRPa (57 years, IQR: 41–70). The length of hospitalization for patients with CA-MDRPa (median 15 days, IQR: 9–33) during this period was also longer and more variable than that of patients with CA-non-MDRPa (median 12 days, IQR: 7–22). Figure 1 (Fig. 1) also shows an increasing trend in the length of hospitalization from 2018 to 2021, regardless of multidrug-resistant status. The proportion of patients with CA-MDRPa upon admission who suffered in-hospital mortality during hospitalization was significantly higher than that of patients with CA-non-MDRPa (8.6% vs. 3.6%, *p*=0.001) (Supplementary material 4 in Attachment 1).

### Analysis of the distribution of CA-MDRPa and CA-non-MDRPa stratified by age and sex

**Figure 2:**
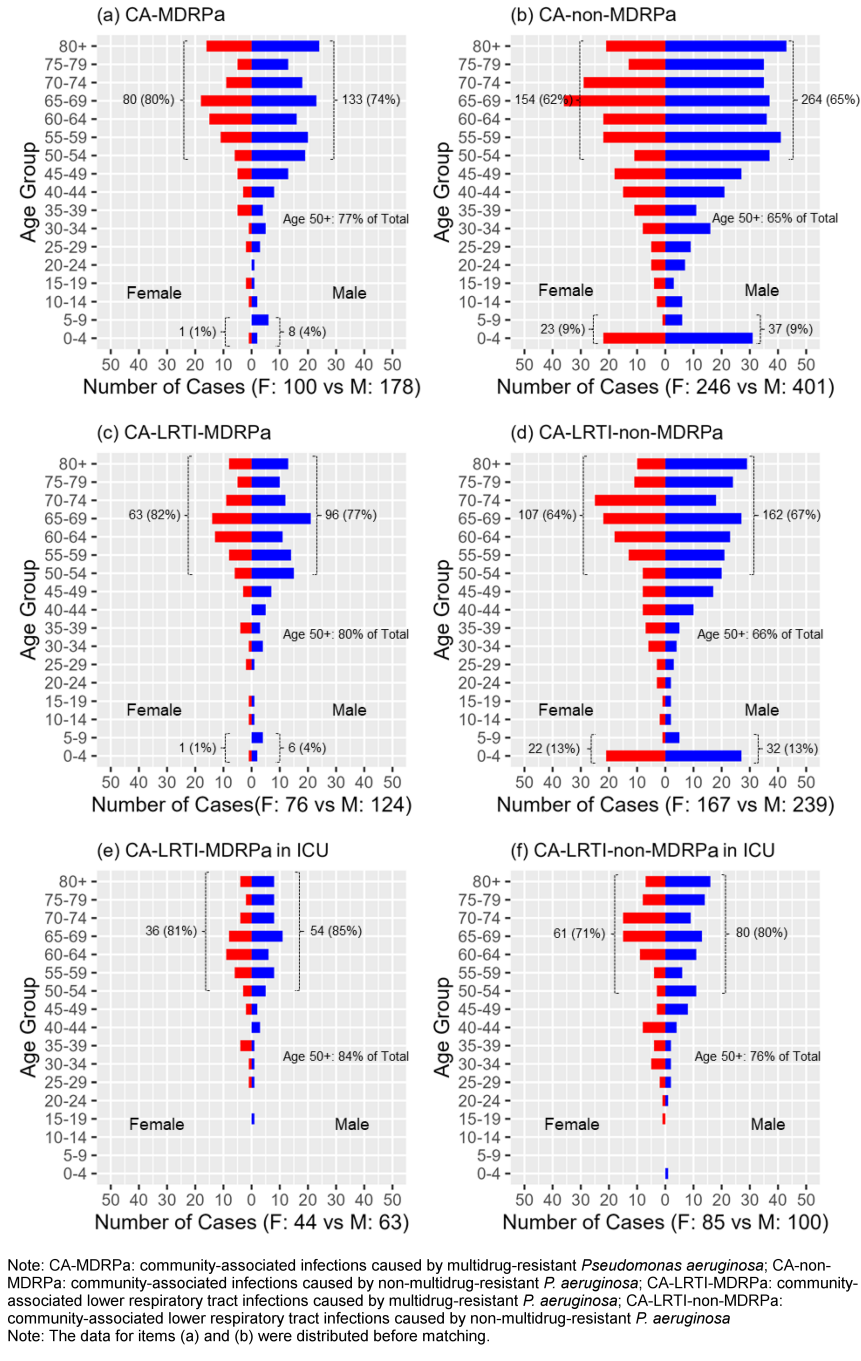
Distributions of CA-MDRPa and CA-non-MDRPa cases stratified by age and sex

Figure 2 (Fig. 2) shows a breakdown of the absolute numbers of cases for both groups, with an analysis of the distribution by age and sex. For the age-specific analysis, the findings indicate that a majority of patients – both those with CA-MDRPa and those with CA-non-MDRPa – were over the age of 50, but with a higher proportion of the CA-MDRPa cases being among the over-50s than the proportion of CA-non-MDRPa cases among the over-50s (77% vs. 65%, *p*<0.001). Furthermore, for the age and sex interaction analysis, the data show that the proportion of CA-MDRPa cases upon admission who were females over the age of 50 was significantly higher than the proportion of CA-non-MDRPa cases who were females over the age of 50 (80% vs. 62%, *p*=0.002). Similarly, the proportion of CA-MDRPa cases upon admission who were males over the age of 50 was significantly higher than the proportion of CA-non-MDRPa cases who were males over the age of 50 (74% vs. 65%, *p*=0.034). In contrast, the proportion of CA-non-MDRPa upon admission who were children aged 0–9 years was higher than the proportion of CA-MDRPa who were children aged 0–9 (9% vs. 3%, *p*=0.001).

### Incidence rate ratios for CA-MDRPa and CA-non-MDRPa 

**Figure 3:**
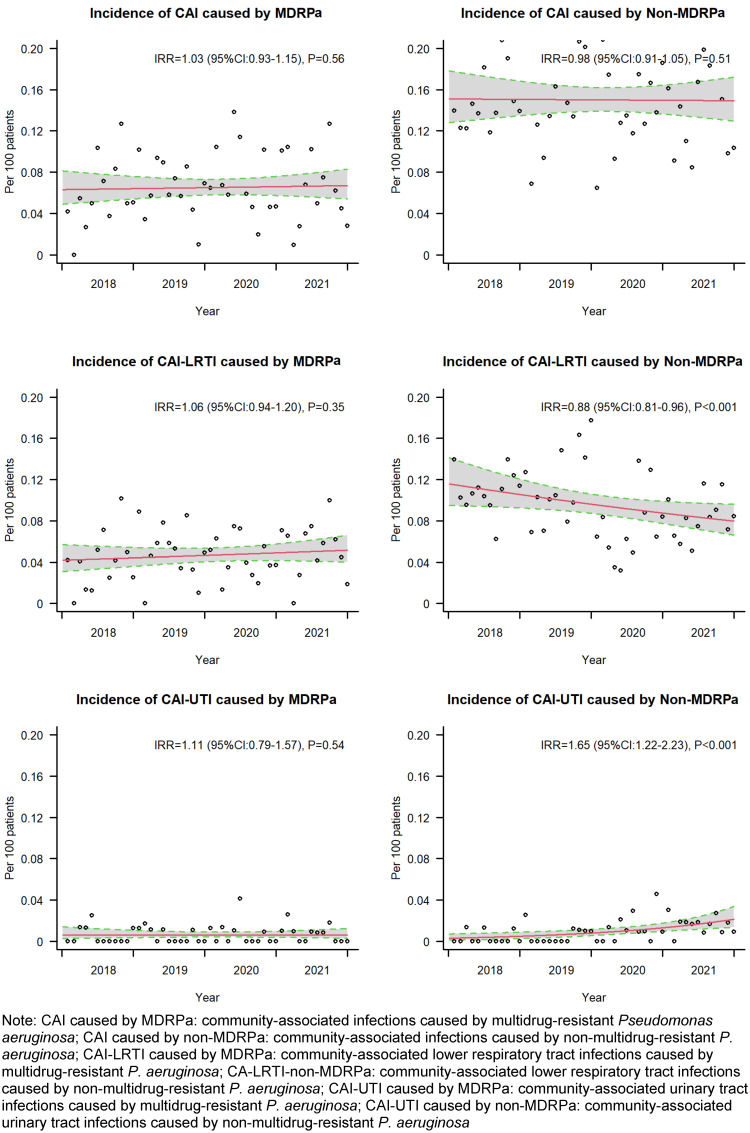
Incidence rate ratios for CA-MDRPa and CA-non-MDRPa

The incidence of diagnosed CA-MDRPa cases showed a slight increase, from 0.060 per 100 admissions in 2018 to 0.065 per 100 admissions in 2021, with an incidence rate ratio (IRR) of 1.03 (95% CI: 0.93–1.15). Conversely, the incidence of CA-non-MDRPa cases slightly decreased, from 0.150 to 0.146 per 100 admissions, with an IRR of 0.98 (95% CI: 0.91–1.05). However, for both groups, the IRRs were not statistically significant, as shown in Figure 3 (Fig. 3).

The analysis of the incidence trends for diagnosed community-associated lower respiratory tract infections revealed distinct patterns: the multidrug-resistant group displayed an upward trend that was not statistically significant, while the non-multidrug-resistant group showed a downward trend that was significant, from 0.109 per 100 admissions in 2018 to 0.083 per 100 admissions in 2021, with an IRR of 0.88 (95% CI: 0.81–0.96). Furthermore, when examining the incidence of diagnosed community-associated urinary tract infections, both groups showed upward trends, but only the trend of the non-multidrug-resistant group was statistically significant, rising from 0.003 to 0.016 per 100 admissions, with an IRR of 1.65 (95% CI: 1.22–2.23).

### Multivariable conditional logistic regression analysis 

**Figure 4:**
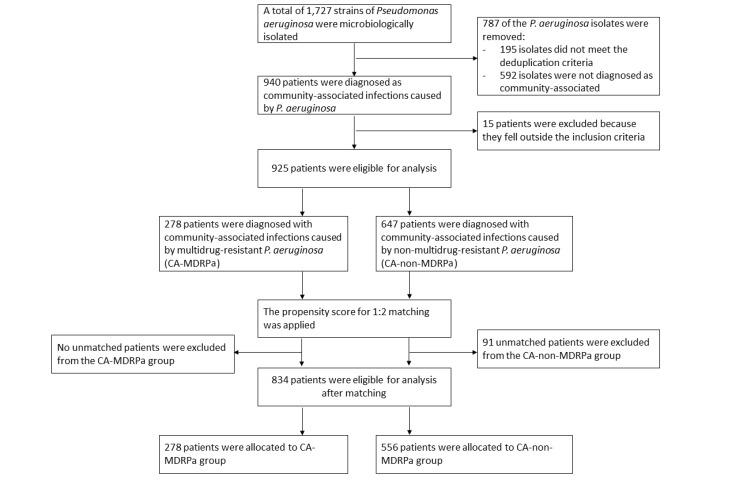
Flow chart of data collection and matching

Figure 4 (Fig. 4) shows the flow chart of the propensity-score matching, which allocated 278 patients to the CA-MDRPa group and 556 patients to the CA-non-MDRPa group. The effectiveness of the matching process is illustrated in the Supplementary material 5 and 6 in Attachment 1. Supplementary material 7 (Attachment 1) presents the frequencies of the covariates in the CA-MDRPa and CA-non-MDRPa matched groups. The conditional regression model demonstrated superiority over the mixed-effects logistic regression model in terms of model evaluation metrics (Table 1 (Tab. 1)). Diagnosis at admission of a neurological disorder (OR: 2.96, 95% CI: 1.62–5.42), cardiovascular disease (OR: 2.34, 95% CI: 1.05–5.21), pulmonary disease (OR: 1.72, 95% CI: 1.01–2.90), or urinary tract infection (OR: 2.93, 95% CI: 1.46–5.88) or cefoperazone/sulbactam use prior to admission (OR: 3.86, 95% CI: 1.04–14.24) was significantly associated with CA-MDRPa upon admission. Furthermore, in a subgroup analysis of patients admitted to the intensive care unit, a diagnosis of lower respiratory tract infection (OR: 5.26, 95% CI: 1.10–25.17) was significantly associated with CA-MDRPa upon admission (Supplementary material 8 in Attachment 1). 

### Clinical outcomes 

From the matched data, a diagnosis of CA-MDRPa upon admission was significantly associated with an increased likelihood of developing ESBL-producing *P. aeruginosa* during hospitalization (OR: 2.70, 95% CI: 1.53–4.77) and with increased in-hospital mortality (OR: 2.24, 95% CI: 1.17–4.28) (Table 2 (Tab. 2)).

## Discussion

Globally, there is limited research in the literature regarding the incidence trends of CA-MDRPa and CA-non-MDRPa, especially during the pandemic [27], [28]. The novelty of this study lies not only in filling a research gap by analyzing incidence trend data for CA-MDRPa, spanning from the pre-COVID to the post-COVID era, but also in providing a detailed epidemiological profile to assist in identifying high-risk patients and factors associated with CA-MDRPa upon admission. The study also provides epidemiological reference data to support the development of in-house admission screening protocols for *P. aeruginosa* at our institution, thereby helping to reduce the risk of patient-to-patient transmission and ultimately ensuring patient safety [29].

We observed a slight but non-significant upward trend in the incidence of CA-MDRPa between 2018 and 2021. This is in line with a study by Lyu et al. who found that the prevalence of MDRPa in Guangzhou, Southern China, increased from 9.4% to 12.6% between 2017 and 2021, without demonstrating statistical significance [30]. Our finding, however, is different than the EARS-Net surveillance report, which showed that between 2016 and 2020, the prevalence of MDRPa in Europe dropped from 15.0% to 12.1% [7]. Our data do not allow to fully identify the underlying causes for these diverging trends between studies in China and Europe, but the contrast becomes particularly relevant when considering the persistent reports of inappropriate use of antibiotics in community and primary care settings in China [31]. Indeed, Yin et al. [32] reported that 10% of participants in a survey conducted just before the start of the COVID pandemic engaged in self-medication with antibiotics, and Zhao et al. [33], used data from the Beijing Data Center for Rational Use of Drugs to show that a significant proportion of outpatient prescriptions (48%) between 2014 and 2018 were inappropriate. It is likely that social phenomena might be responsible for promoting CA-MDRPa in our hospital as in other Chinese hospitals.

We also observed a trend of fewer community-associated lower respiratory tract infections caused by non-MDRPa upon admission, which is encouraging and may be indicative of the effectiveness of the extensive COVID-19 infection-control measures in the community (e.g., wearing masks, practicing hand hygiene with alcohol-based hand rubs, and household environmental disinfection), which significantly reduced the likelihood of CA-non-MDRPa among generally admitted patients [30]. However, there was also a significant upward trend in community-associated urinary tract infections caused by non-MDRPa upon admission, which might be directly associated with *P. aeruginosa* resistance to fluoroquinolones, indirectly evidenced by the detection of large quantities of fluoroquinolones in municipal effluent [34]. There may thus be a risk of fluoroquinolone resistance being transmitted to humans through the One Health ecological interaction system, which could manifest as an increase in the incidence of community-associated urinary tract infections caused by quinolone resistance in *P. aeruginosa* [35].

Stratifying our data by sex alone, we observed that total absolute number of CA-MDRPa cases was higher in males than in females. This is consistent with findings from Austria, Germany, and Switzerland, as documented in the EARS-Net surveillance report [7]. Furthermore, when stratifying the data by age, we found that 68% of cases of *P. aeruginosa* fell within the 50–80 age range, which is again consistent with findings from the same countries [7]. However, unlike our analysis, that report did not examine the data by age and sex simultaneously, nor did it consider factors such as infection site and hospital department. Our analysis, however, did include these considerations and suggests that it is important to regularly screen patients older than 50 for *P. aeruginosa* upon admission, regardless of their susceptibility to multidrug resistance [17]. This strategy may be crucial for preventing potential carriers of resistant *P. aeruginosa* pathogens from transmitting the infection to uninfected patients who are not otherwise at risk of carrying resistant strains of *P. aeruginosa*.

Patients admitted with neurological conditions and cardiovascular disease were positively associated with CA-MDRPa upon admission in our analysis. This may be because our medical institution has seen an increase in the volume of specialized treatment services for cardiovascular and cerebrovascular specialties and has expanded its services for referrals from other outpatient centers for patients requiring neurosurgery and care for both cardiovascular and cerebrovascular conditions. Consequently, patients carrying CA-MDRPa are likely to be first identified in the relevant specialist wards. We also observed that being admitted with either a pulmonary disease or a respiratory disorder was also positively associated with CA-MDRPa upon admission, although this is in contrast to a recent systematic review that found that diagnosis of a chronic obstructive pulmonary disease was not associated with a risk of acquiring an MDRPa infection [1]. 

Furthermore, we noted that cefoperazone/sulbactam use prior to admission was significantly associated with CA-MDRPa upon admission. This is consistent with evidence from a longitudinal study conducted in a Beijing hospital that showed that cases of cefoperazone/sulbactam-resistant *P. aeruginosa* infection in the emergency department significantly increased year-over-year from 2013 to 2017 [36]. Unlike other antibiotics, cefoperazone/sulbactam, which is commonly used to treat lower respiratory tract infections and urinary tract infections, is a restricted-use antibiotic, and its prescription is authorized by director-level physicians in outpatient clinics [4]. It is therefore imperative, when managing referred patients, for physicians and infection-control specialists to thoroughly review their medical records, paying particular attention to the use of broad-spectrum cephalosporins. It is also crucial to be careful about the onset of CA-MDRPa upon admission and to screen for patients with a documented history of broad-spectrum cephalosporin use. A systematic review found a significant association between the acquisition of carbapenem-resistant *P. aeruginosa* infection and previous use of piperacillin-tazobactam [1]. Our study did not observe such an association, which is encouraging and might indicate partial success of the efforts of China’s National Action Plan to Contain Antimicrobial Resistance [4].

From a clinical and infection-control perspective, we observed an association between CA-MDRPa upon admission and the subsequent development of ESBL-producing *P. aeruginosa* during hospitalization. Hospital outbreaks of MDRPa are often caused by *P. aeruginosa* clones that produce metallo-β-lactamases (MBLs) [37], and both MBLs and ESBLs are enzymes that confer bacterial resistance to β-lactam antibiotics. Patients carrying MBL-producing *P. aeruginosa* could introduce the pathogen into the hospital environment upon admission, particularly into ward sink drains, which can then act as reservoirs [38]. If hospital’s environmental hygiene strategies are not sufficiently robust, the use of broad-spectrum antibiotics in critically ill patients could further select for *Enterobacteriaceae* that produce ESBLs and MBLs in the hospital environment during daily medical contact [39], making infections increasingly difficult to treat. Environmental hygiene – as well as infection-control strategies – should therefore be strengthened at the hospital level.

The main strength of this study is that it provides a hospital-wide, large-sample, longitudinal analysis spanning the COVID-19 period. It offers a comprehensive overview of the incidence data of CA-MDRPa and CA-non-MDRPa, which, until now, have not been documented within the infection-control community in Southern China. Furthermore, propensity-score matching was used to ensure that the CA-MDRPa and CA-non-MDRPa groups were demographically well-balanced so that the most comparable cases would be used to estimate the effects of covariates on the outcomes within the two groups. Finally, the conditional regression model demonstrated superiority over and greater robustness than a mixed-effect logistic regression model by allowing for pairing or stratification, thereby reducing bias and producing more accurate estimates. 

However, this study also has limitations. First, the study was conducted in a tertiary-care hospital that serves as a referral center for more severely ill patients and may therefore not be representative of primary and secondary medical centers. Second, a recent systematic review has highlighted a correlation between MDRPa and respiratory tract infections, suggesting a potential rise in co-infection leading to bacteremia [27], but our data structure did not allow for an analysis of this relationship. That finding should alert physicians treating patients with community-associated respiratory tract infections caused by MDRPa to the possibility of further bacteremia, especially in patients in intensive care units (Supplementary material 8 in Attachment 1). Third, our data did not include patients’ histories of antimicrobial use (other than cefoperazone/sulbactam and piperacillin-tazobactam) due to limitations in documentation. We therefore could not assess associations between CA-MDRPa and the consumption of other antibiotics prior to admission, unlike the systematic review [1].

## Conclusions

We observed a non-significant increase in the incidence of CA-MDRPa between 2018 and 2021, a significant decrease in CA-non-MDRPa for respiratory tract infections, and a significant increase in CA-non-MDRPa for urinary tract infections. Higher CA-MDRPa rates were observed among older patients, emphasizing the need for regular admissions screening for these individuals. Diagnoses of neurological conditions, cardiovascular diseases, respiratory disorders, and urinary tract infections, and cefoperazone/sulbactam use prior to admission were risk factors associated with having CA-MDRPa upon admission. These findings underscore the importance of targeted infection-control screening strategies and the need for continuous surveillance to mitigate the impact of multidrug-resistant infections.

## Notes

### Competing interests

The authors declare that they have no competing interests.

### Authors’ ORCIDs

- Jiayao Lei: 0000-0002-4718-1414
- Evangelos I. Kritsotakis: 0000-0002-9526-3852
- Jiancong Wang: 0000-0001-6284-9702

### Ethical approval

This study received ethical approval from the Ethics Committee of Dongguan Songshan Lake Tungwah Hospital (Reference number: SDHKY-2024-005-01). Because the data originated from the local nosocomial surveillance system and did not include any individually identifying patient information, written informed consent from patients was not required.

### Funding sources

None

### Data protection statement

Due to data protection reasons, the clinical patient-level datasets generated and/or analyzed during the current study cannot be made publicly available or shared with any third parties/researchers who are not part of the current research group. The datasets are only available upon reasonable request, provided that their release is consistent with the consent given by the ethical approval from the Chinese Clinical Research Ethics Committees. Additionally, the data transfer agreement from the legal department of the Dongguan Tungwah Hospitals must be accepted.

### Acknowledgements

The authors would like to thank the Medical Review & Ethics Committee of Dongguan Songshan Lake Tungwah Hospital for granting permission to publish this paper. We would also like to extend our appreciation and thanks to the frontline infection control physicians and nurses in our hospital who did an excellent job in infection control, ensuring patient safety.

## Supplementary Material

Clinical burden of community-associated infections caused by multidrug-resistant Pseudomonas aeruginosa: A propensity-matched longitudinal cohort study in Southern China

## Figures and Tables

**Table 1:**
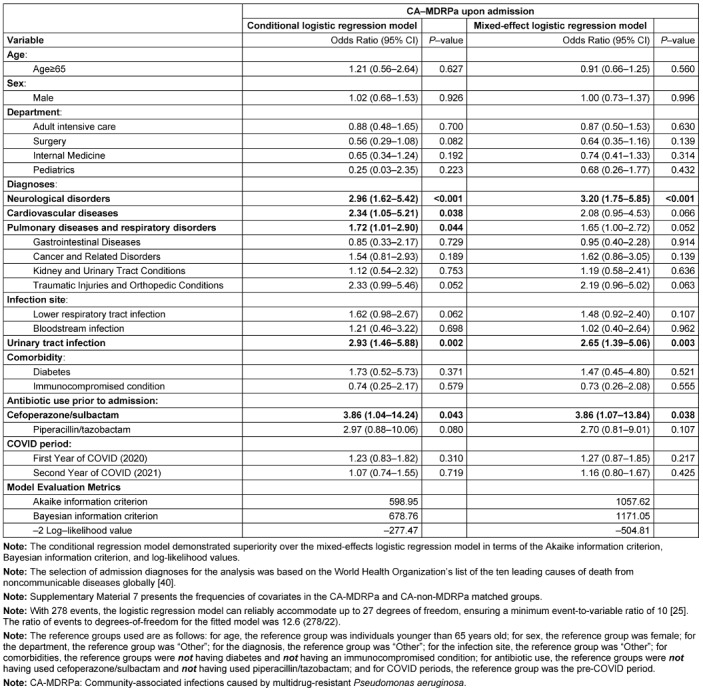
Multivariable analysis of clinical factors associated with CA-MDRPa upon admission using conditional and mixed effects logistic regression models in the propensity-score-matched dataset (n=834 observations)

**Table 2:**
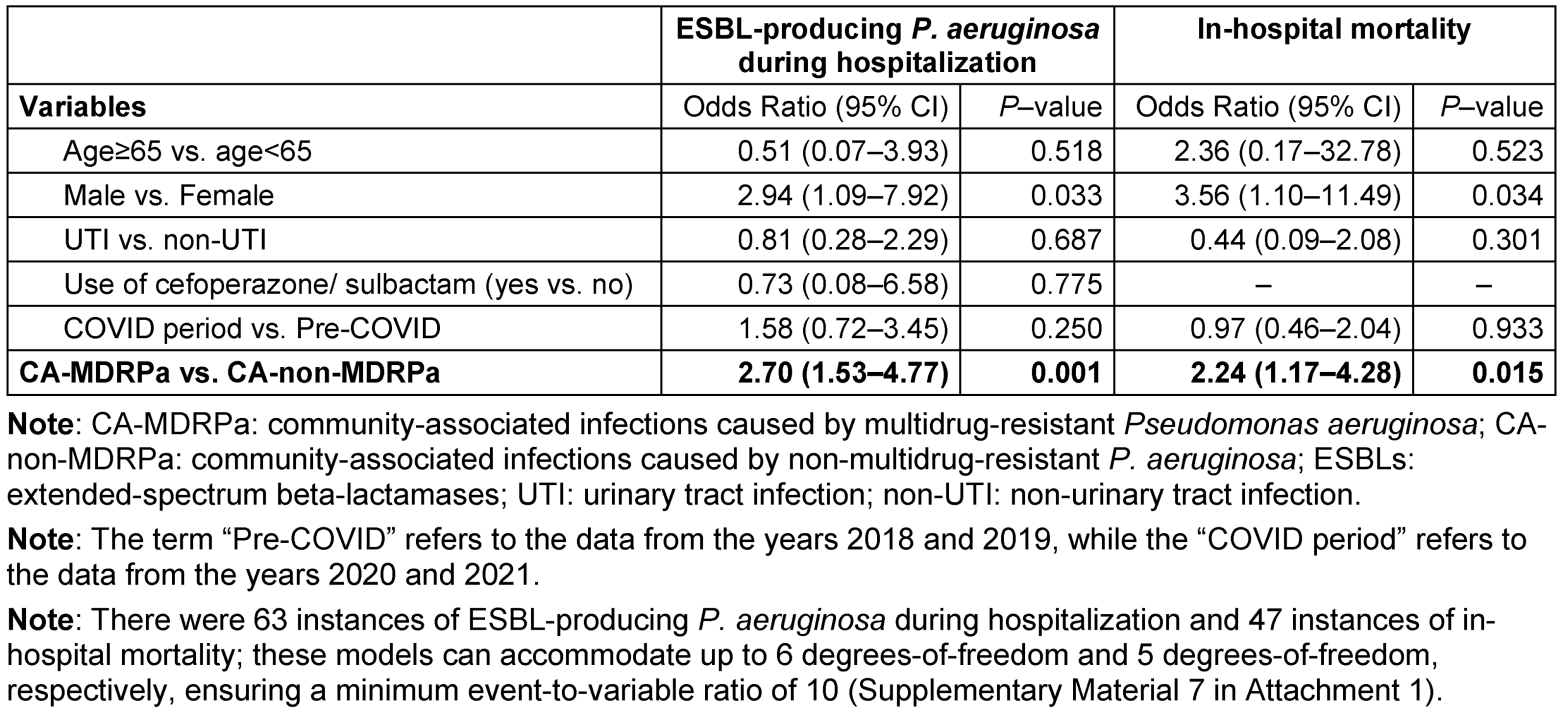
Multivariable conditional logistic regression analysis of a propensity-score-matched data to examine the impact of CA-MDRPa upon admission (as an independent covariate) on clinical outcomes during hospitalization (n=834 observations)
