# Supplementary material for: Clinical burden of community-associated infections caused by multidrug-resistant Pseudomonas aeruginosa: a propensity-matched longitudinal cohort study in Southern China
Source: GMS Hyg Infect Control. 2024 Oct 23;19:Doc51. doi: 10.3205/dgkh000506 (PMC11565634; doi:10.3205/dgkh000506)
Supplement: Clinical burden of community-associated infections caused by multidrug-resistant Pseudomonas aeruginosa: A propensity-matched longitudinal cohort study in Southern China [file HIC-19-51-s-001.pdf]

Attachment 1

Clinical burden of community-associated infections caused by multidrug-resistant *Pseudomonas aeruginosa*: A propensity-matched longitudinal cohort study in Southern China

Supplementary Material:

Supplementary Material 1. R code for the propensity-score matching

Supplementary Material 2. R code for the multivariable analysis using conditional and mixed effect logistic regression models

Supplementary Material 3. STROBE Statement—Checklist of items that should be included in reports of cohort studies

Supplementary Material 4. Frequencies of covariates in the CA-MDRPa and CA-non-MDRPa groups *before* matching – a total of 925 observations

Supplementary Material 5. Propensity score distributions

Supplementary Material 6. Summary of the balance for the matched data

Supplementary Material 7. Frequencies of covariates in the CA-MDRPa and CA-non-MDRPa groups *after* matching – a total of 834 observations

Supplementary Material 8. Multivariable analysis of clinical factors associated with CA-MDRPa upon admission using conditional logistic regression models in a propensity-score-matched dataset for a subgroup analysis of patients admitted to the intensive care unit

Attachment to: Zhou M, Xu B, Guo Z, Zeng Y, Lei J, Kritsotakis EI, Wang J. Clinical burden of community-associated infections caused by multidrug-resistant Pseudomonas aeruginosa: a propensity-matched longitudinal cohort study in Southern China. GMS Hyg Infect Control. 2024;19:Doc51. DOI: 10.3205/dgkh000506

Supplementary Material 1. R code for the propensity-score matching

#Step 1: Creating propensity score

```
library(MatchIt)

formula          <- mdr ~ age + gender + admission_department

propensity_model <- glm(formula, family = binomial(), data = df)

summary(propensity_model)

# Obtain the propensity score

df$propensity_score <- predict(propensity_model, type = "response")

# Splitting the data based on 'mdr' values

df_mdr_1 <- df[df$mdr == 1, ]

df_mdr_0 <- df[df$mdr == 0, ]

# Plotting the densities

png(filename=output_file1, width=600, height=600)

plot(density(df_mdr_1$propensity_score), col="red", main="Before matching", xlab="Propensity Score", cex.main=1.5)

lines(density(df_mdr_0$propensity_score), col="blue")

legend("topright", legend=c("MDRPa", "non-MDRPa"), fill=c("red", "blue"))
```

#Step 2: Matching

```
# Using MatchIt for matching based on the estimated propensity scores

matchit_model <- matchit(formula,  data=df,  distance='logit', method='nearest', replace=FALSE, caliper=0.2, discard=, ratio=2)

# Checking the balance after matching

summary(matchit_model, standardize = TRUE)

# Extract the matched data

matched_data <- match.data(matchit_model)

# Splitting the data based on 'mdr' values

matched_data_mdr_1 <- matched_data[matched_data$mdr == 1, ]

matched_data_mdr_0 <- matched_data[matched_data$mdr == 0, ]

# Plotting the densities

png(filename=output_file2, width=600, height=600)

plot(density(matched_data_mdr_1$propensity_score), col="red", main="After matching", xlab="Propensity Score", cex.main=1.5)

lines(density(matched_data_mdr_0$propensity_score), col="blue")

legend("topright", legend=c("MDRPa", "non-MDRPa"), fill=c("red", "blue"))
```

Supplementary Material 2. R code for the multivariable analysis using conditional and mixed effect logistic regression models

```
# Conditional logistic regression

library(survival)

Model_1 <- clogit(mdr ~ age65 + sex + admission_department + admission_diagnosis +
                  infection_site + diabetes + immunocompromised + use_cefoperazone_sulbactam +
                  use_piperacillin_tazobactam + COVID_period + strata(subclass), data = matched_data)

summary(Model_1)


# Mixed effects logistic regression

library(lme4)

Model_2 <- glmer(mdr ~ age65 + sex + admission_department + admission_diagnosis +
                 infection_site + diabetes + immunocompromised + use_cefoperazone_sulbactam +
                 use_piperacillin_tazobactam + COVID_period + (1|subclass), data = matched_data, family = binomial)

summary(Model_2)

library(broom.mixed)

m2<-tidy(Model_2,conf.int=TRUE,exponentiate=TRUE,effects="fixed")

print(m2, n=Inf)
```

Supplementary Material 3. STROBE Statement—Checklist of items that should be included in reports of cohort studies [1]

Study title: Incidence, outcomes, and clinical factors associated with community-associated infections caused by multidrug-resistant Pseudomonas aeruginosa among hospitalized patients in Southern China: A propensity-matched longitudinal cohort study

	Item No	Recommendation	Page(s)
**Title and abstract**	1	(*a*) Indicate the study's design with a commonly used term in the title or the abstract	1, 2
		(*b*) Provide in the abstract an informative and balanced summary of what was done and what was found	2
Introduction			
Background/rationale	2	Explain the scientific background and rationale for the investigation being reported	3-4
Objectives	3	State specific objectives, including any prespecified hypotheses	4
Methods			
Study design	4	Present key elements of study design early in the paper	5
Setting	5	Describe the setting, locations, and relevant dates, including periods of recruitment, exposure, follow-up, and data collection	5
Participants	6	(*a*) Give the eligibility criteria, and the sources and methods of selection of participants. Describe methods of follow-up	5-6
		(*b*) For matched studies, give matching criteria and number of exposed and unexposed	7
Variables	7	Clearly define all outcomes, exposures, predictors, potential confounders, and effect modifiers. Give diagnostic criteria, if applicable	7-8
Data sources/ measurement	8	For each variable of interest, give sources of data and details of methods of assessment (measurement). Describe comparability of assessment methods if there is more than one group	7-8
Bias	9	Describe any efforts to address potential sources of bias	7-8
Study size	10	Explain how the study size was arrived at	We included all data
Quantitative variables	11	Explain how quantitative variables were handled in the analyses. If applicable, describe which groupings were chosen and why	6-8
Statistical methods	12	(*a*) Describe all statistical methods, including those used to control for confounding	6-8
		(*b*) Describe any methods used to examine subgroups and interactions	8
		(*c*) Explain how missing data were addressed	No missing data; 8

		(*d*) If applicable, explain how loss to follow-up was addressed	Not applicable
		(*e*) Describe any sensitivity analyses	Not applicable
Results			
Participants	13	(a) Report numbers of individuals at each stage of study—e.g., numbers potentially eligible, examined for eligibility, confirmed eligible, included in the study, completing follow-up, and analysed	10-11
		(b) Give reasons for non-participation at each stage	Not applicable
		(c) Consider use of a flow diagram	32; Figure 4
Descriptive data	14	(a) Give characteristics of study participants (e.g., demographic, clinical, social) and information on exposures and potential confounders	10-11, Supplementary material 4
		(b) Indicate number of participants with missing data for each variable of interest	No missing data
		(c) Summarise follow-up time (e.g., average and total amount)	Not applicable
Outcome data	15	Report numbers of outcome events or summary measures over time	10, Supplementary material 4
Main results	16	(*a*) Give unadjusted estimates and, if applicable, confounder-adjusted estimates and their precision (e.g., 95% confidence interval). Make clear which confounders were adjusted for and why they were included	10-12, Table 1 and Table 2
		(*b*) Report category boundaries when continuous variables were categorized	Not applicable
		(*c*) If relevant, consider translating estimates of relative risk into absolute risk for a meaningful time period	Not applicable
Other analyses	17	Report other analyses done—e.g., analyses of subgroups and interactions, and sensitivity analyses	Supplementary material 8
Discussion			
Key results	18	Summarise key results with reference to study objectives	13-15
Limitations	19	Discuss limitations of the study, taking into account sources of potential bias or imprecision. Discuss both direction and magnitude of any potential bias	16
Interpretation	20	Give a cautious overall interpretation of results considering objectives, limitations, multiplicity of analyses, results from similar studies, and other relevant evidence	13-16
Generalisability	21	Discuss the generalisability (external validity) of the study results	16
Other information			
Funding	22	Give the source of funding and the role of the funders for the present study and, if applicable, for the original study on which the present article is based	17

Supplementary Material 4. Frequencies of covariates in the CA-MDRPa and CA-non-MDRPa groups ***before*** matching – a total of 925 observations

Variable	Total, *N*	Total, *%*	CA-MDRPa, *N*	(%)	CA-non-MDRPa, *N*	(%)
**Total**	**925**		**278**		**647**	
**Age**						
>65	327	35.4%	110	39.6%	217	33.5%
<=65	598	64.6%	168	60.4%	430	66.5%
**Sex**						
Male	579	62.6%	178	64.0%	401	62.0%
Female	346	37.4%	100	36.0%	246	38.0%
**Admission Departments**						
Adult intensive care	326	35.2%	115	41.4%	211	32.6%
Surgery	229	24.8%	58	20.9%	171	26.4%
Internal Medicine	211	22.8%	62	22.3%	149	23%
Paediatrics	73	7.9%	10	3.6%	63	9.7%
Other	86	9.3%	33	11.9%	53	8.2%
**Admission Diagnoses**						
Neurological Disorders	74	8.0%	38	13.7%	36	5.6%
Cardiovascular Diseases	39	4.2%	14	5.0%	25	3.9%
Pulmonary Diseases/Respiratory Disorders	365	39.5%	115	41.4%	250	38.6%
Gastrointestinal Diseases	41	4.4%	8	2.9%	33	5.1%
Cancer and Related Disorders	73	7.9%	23	8.3%	50	7.7%
Kidney and Urinary Tract Conditions	60	6.5%	18	6.5%	42	6.5%
Traumatic Injuries and Orthopaedic Conditions	38	4.1%	13	4.7%	25	3.9%
Others	235	25.4%	49	17.6%	186	28.7%
**Infection sites**						
Lower respiratory tract infection	607	65.6%	201	72.3%	406	62.8%
Bloodstream infection	35	3.8%	7	2.5%	28	4.3%
Urinary tract infection	74	8.0%	29	10.4%	45	7.0%

Others	209	22.6%	41	14.7%	168	26%
**Diabetes**						
Yes	15	1.6%	5	1.8%	10	1.5%
No	910	98.4%	273	98.2%	637	98.5%
**Immunocompromised condition**						
Yes	21	2.3%	6	2.2%	15	2.3%
No	904	97.7%	272	97.8%	632	97.7%
**Used cefoperazone/sulbactam prior to admission**						
Yes	16	1.7%	8	2.9%	8	1.2%
No	909	98.3%	270	97.1%	639	98.8%
**Used piperacillin/tazobactam prior to admission**						
Yes	14	1.5%	8	2.9%	6	0.9%
No	911	98.5%	270	97.1%	641	99.1%
**COVID Periods (N=834)**						
Second Year of COVID (2021)	264	28.5%	81	29.1%	183	28.3%
First Year of COVID (2020)	235	25.4%	77	27.7%	158	24.4%
Pre-COVID Period (2018-2019)	426	46.1%	120	43.2%	306	47.3%
**Prior to having multidrug resistance**						
Yes	12	1.3%	12	4.3%	0	0
No	913	98.7%	266	95.7%	647	100%
**Development of pandrug-resistant during hospitalization**						
Yes	4	0.4%	4	1.4%	0	0
No	921	99.6%	274	98.6%	647	100%
**Development of ESBLs during hospitalization**						
Yes	64	6.9%	33	11.9%	31	4.8%
No	861	93.1%	245	88.1%	616	95.2%
**Clinical outcomes**						
In-hospital mortality	47	5.1%	24	8.6%	23	3.6%
Discharge alive	878	94.9%	254	91.4%	624	96.4%

Supplementary Material 5. Propensity score distributions

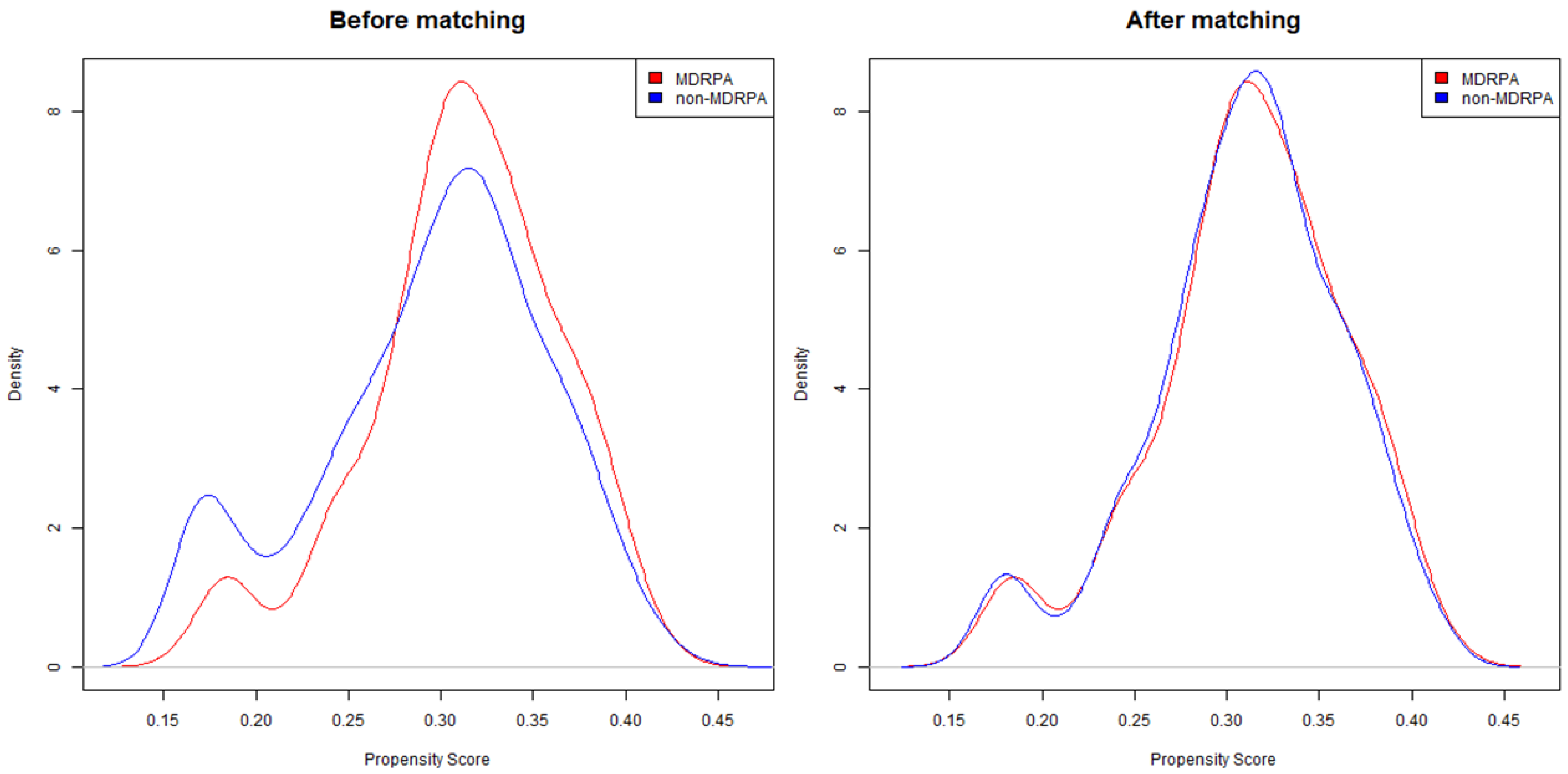

Note: For detailed information, please refer to Supplementary Material 6.

Note: The red line represented the propensity score indexed for community-associated infections caused by multidrug-resistant *Pseudomonas aeruginosa*, while the blue line represented the propensity score indexed for community-associated infections caused by non-multidrug-resistant *P. aeruginosa*.

Attachment to: Zhou M, Xu B, Guo Z, Zeng Y, Lei J, Kritsotakis EI, Wang J. Clinical burden of community-associated infections caused by multidrug-resistant Pseudomonas aeruginosa: a propensity-matched longitudinal cohort study in Southern China. GMS Hyg Infect Control. 2024;19:Doc51. DOI: 10.3205/dgkh000506

Supplementary Material 6. Summary of the balance for the matched data

	Before Propensity Matching			After Propensity Matching		
**Variables**	Standardized difference	eCDF Mean	eCDF Max	Standardized difference	eCDF Mean	eCDF Max
Age	0.3284	0.0661	0.1361	0.0374	0.0092	0.0414
Gender	0.0427	0.0205	0.0205	-0.0112	0.0054	0.0054
Admission departments	-0.0800	0.0362	0.0875	0.0000	0.0266	0.0665

Note: The standardized difference is calculated to assess the relative imbalance between the exposed and unexposed groups, with values of less than 0.2 considered to be adequately balance.

Note: eCDF Mean: Empirical Cumulative Distribution Function Mean. This measures the maximum difference in the empirical cumulative distribution functions between the two groups for each variable.

Note: eCDF Max: Empirical Cumulative Distribution Function Maximum. This measures the maximum difference in the empirical cumulative distribution functions between the two groups for each variable.

Supplementary Material 7. Frequencies of covariates in the CA-MDRPa and CA-non-MDRPa groups ***after*** matching – a total of 834 observations

**Variable**		**Total, *N***	**Total, *%***	**CA-MDRPa, *N***	**(%)**	**CA-non-MDRPa, *N***	**(%)**
**Total**		**834**		**278**		**556**	
**Age**							
	>65	326	39.1%	110	39.6%	216	38.8%
	<=65	508	60.9%	168	60.4%	340	61.2%
**Sex**							
	Male	537	64.4%	178	64.0%	359	64.6%
	Female	297	35.6%	100	36.0%	197	35.4%
**Admission Departments**							
	Adult intensive care	308	36.9%	115	41.4%	193	34.7%
	Surgery	215	25.8%	58	20.9%	157	28.2%
	Internal Medicine	198	23.7%	62	22.3%	136	24.5%
	Paediatrics	31	3.7%	10	3.6%	21	3.8%
	Other	82	9.8%	33	11.9%	49	8.8%
**Admission Diagnoses**							
	Neurological Disorders	73	8.8%	38	13.7%	35	6.3%
	Cardiovascular Diseases	37	4.4%	14	5.0%	23	4.1%
	Pulmonary Diseases/Respiratory Disorders	310	37.2%	115	41.4%	195	35.1%
	Gastrointestinal Diseases	39	4.7%	8	2.9%	31	5.6%
	Cancer and Related Disorders	69	8.3%	23	8.3%	46	8.3%
	Kidney and Urinary Tract Conditions	58	7.0%	18	6.5%	40	7.2%
	Traumatic Injuries and Orthopaedic Conditions	34	4.1%	13	4.7%	21	3.8%
	Others	214	25.7%	49	17.6%	165	29.7%
**Infection sites**							
	Lower respiratory tract infection	543	65.1%	201	72.3%	342	61.5%
	Bloodstream infection	32	3.8%	7	2.5%	25	4.5%
	Urinary tract infection	69	8.3%	29	10.4%	40	7.2%

Others	190	22.8%	41	14.7%	149	26.8%
**Diabetes**						
Yes	15	1.8%	5	1.8%	10	1.8%
No	819	98.2%	273	98.2%	546	98.2%
**Immunocompromised condition**						
Yes	21	2.5%	6	2.2%	15	2.7%
No	813	97.5%	272	97.8%	541	97.3%
**Used cefoperazone/sulbactam prior to admission**						
Yes	12	1.4%	8	2.9%	4	0.7%
No	822	98.6%	270	97.1%	552	99.3%
**Used piperacillin/tazobactam prior to admission**						
Yes	13	1.6%	8	2.9%	5	0.9%
No	821	98.4%	270	97.1%	551	99.1%
**COVID Periods (N=834)**						
Second Year of COVID (2021)	240	28.8%	81	29.1%	159	28.6%
First Year of COVID (2020)	218	26.1%	77	27.7%	141	25.4%
Pre-COVID Period (2018-2019)	376	45.1%	120	43.2%	256	46.0%
**Prior to having multidrug resistance**						
Yes	12	1.4%	12	4.3%	0	0%
No	822	98.6%	266	95.7%	556	100%
**Development of pandrug-resistant during hospitalization**						
Yes	4	0.5%	4	1.4%	0	0
No	830	99.5%	274	98.6%	556	100%
**Development of ESBLs during hospitalization**						
Yes	63	7.6%	33	11.9%	30	5.4%
No	771	92.4%	245	88.1%	526	94.6%
**Clinical outcomes**						
In-hospital mortality	47	5.6%	24	8.6%	23	4.1%
Discharge alive	787	94.4%	254	91.4%	533	95.9%

Supplementary Material 8. Multivariable analysis of clinical factors associated with CA-MDRPa upon admission using conditional logistic regression models in a propensity-score-matched dataset for a subgroup analysis of patients admitted to the intensive care unit

	**CA-MDRPa upon admission**	
**Variables**	Odds Ratio (95% CI)	*P*-value
**Diagnoses**:		
Pulmonary Diseases and Respiratory Disorders	1.37 (0.70-2.68)	0.351
**Infection sites**:		
**Lower respiratory tract infection**	**5.26 (1.10-25.17)**	**0.038**
Bloodstream infection	1.96 (0.18-21.94)	0.585
Urinary tract infection	15.5 (0.93-257.58)	0.056
**Comorbidity**:		
Immunocompromised condition	0.21 (0.01-5.13)	0.335
**Antibiotic use prior to admission:**		
Cefoperazone/sulbactam	0.97 (0.07-14.16)	0.981
Piperacillin/tazobactam	2.41 (0.22-26.48)	0.471
**COVID-periods:**		
First Year of COVID (2020)	2.16 (0.93-5.02)	0.073
Second Year of COVID (2021)	1.19 (0.56-2.53)	0.656

Note: The reference groups used are as follows: for the diagnosis, the reference group was ***not*** diagnosing as pulmonary diseases and respiratory disorders; for the infection site, the reference group was “Other”; for comorbidity, the reference group was ***not*** having an immunocompromised condition; for antibiotic use, the reference groups were ***not*** having used cefoperazone/sulbactam and ***not*** having used piperacillin/tazobactam; and for COVID periods, the reference group was the pre-COVID period.

Reference

[1] von Elm E, Altman DG, Egger M, et al. Strengthening the Reporting of Observational Studies in Epidemiology (STROBE) statement: guidelines for reporting observational studies. Bmj. 2007;335:806-8. DOI: 10.1136/bmj.39335.541782.AD
